# KRAS as a Key Oncogene in the Clinical Precision Diagnosis and Treatment of Pancreatic Cancer

**DOI:** 10.7150/jca.76695

**Published:** 2022-08-31

**Authors:** Manxiong Dai, Shaofeng Chen, Xiong Teng, Kang Chen, Wei Cheng

**Affiliations:** 1 Department of Hepatobiliary Surgery, Hunan Provincial People's Hospital, The First Affiliated Hospital of Hunan Normal University, Changsha, 410005 Hunan Province, China.; 2 Xiangyue Hospital Affiliated to Hunan Institute of Parasitic Diseases, National Clinical Center for Schistosomiasis Treatment, Yueyang 414000, Hunan Province, China.; 3 Translational Medicine Laboratory of Pancreas Disease of Hunan Normal University, Changsha 410005, China.

**Keywords:** Pancreatic neoplasms, KRAS gene, Targeted therapy, Diagnosis, Review

## Abstract

Pancreatic ductal adenocarcinoma (PDAC) is one of the most malignant tumors, with a 5-year survival rate of less than 10%. At present, the comprehensive treatment based on surgery, radiotherapy and chemotherapy has encountered a bottleneck, and targeted immunotherapy turns to be the direction of future development. About 90% of PDAC patients have KRAS mutations, and KRAS has been widely used in the diagnosis, treatment, and prognosis of PDAC in recent years. With the development of liquid biopsy and gene testing, KRAS is expected to become a new biomarker to assist the stratification and prognosis of PDAC patients. An increasing number of small molecule inhibitors acting on the KRAS pathway are being developed and put into the clinic, providing more options for PDAC patients.

## Introduction

In 2020, there were 490,000 new cases of pancreatic cancer and more than 460,000 deaths worldwide 1, with a 5-year survival rate less than 10% 2. Pancreatic cancer, a serious disease threatening the life and health of people, is projected to become the second leading cause of cancer death worldwide by 2030 3. Despite the continuous development of surgical techniques and the emergence of various chemotherapies for PDAC, the improvement in patient survival is still rather limited.

In 2008, Jones et al. 4 conducted the first full exon sequencing of pancreatic cancer, with different gene mutations in 12 tumor-related pathways observed. Subsequent studies confirmed these findings and identified four major driver mutations including KRAS, tumor protein p53 (TP53), SMAD family member 4 (SMAD4) and cyclin dependent kinase inhibitor 2A (CDKN2A) 5, among which, KRAS mutation is the most common one, with a mutation rate up to 90%. However, how KARS mutation plays its clinical value in the clinical diagnosis and treatment of PDAC and whether it has the possibility of becoming a molecular targeted therapy site to improve the diagnosis and treatment of PDAC are all questions being frequently explored in the field of precision diagnosis and treatment of pancreatic tumors. Against such a context, the focus of this paper will be placed on the occurrence of KRAS mutations in PDAC, types of KRAS mutations and their correlation with the clinical prognosis of PDAC, and the research progress in precision diagnosis and treatment related to KRAS mutations.

## 1. The Oncogenic Mechanism of KRAS Gene Mutation and Its Relationship with Pancreatic Cancer

KRAS mutations are the most common mutations in human solid tumors 6, and can be found in 90% of pancreatic cancer patients 7. Similar alternative mutations activating RAS downstream pathways are detected in another 10% of KRAS wild-type patients 8. KRAS is a subtype of the RAS gene family, which also includes HRAS and NRAS. KRAS gene is located on human chromosome 12, encoding KRAS4A and KRAS4B proteins, also known as P12 protein due to its molecular weight of 12KDa^9^. KRAS protein is essentially a GTP enzyme, activated when binding to GTP and activating more than 80 downstream effector proteins and multiple pathways including rapidly accelerated fibrosarcoma (RAF)- mitogen activated protein kinase (MEK)- extracellular signal regulated kinase (ERK), phosphatidylinositol 3 kinase (PI3K)- protein kinase B (AKT)- mammalian target of rapamycin (mTOR), Hippo-yes associated protein (YAP), etc. KRAS protein is inactivated when binding to GDP. The conversion process of KRAS protein between its activated and inactivated states is regulated by two types of factors, i.e., guanine nucleotide exchange factor (GEF) and GTP-activated protein (GAP). The former promotes the binding of KRAS and GTP to activate KRAS, while the latter hydrolyzes GTP for the inactivation of KRAS 10. Epidermal growth factor receptor (EGFR) upstream of RAS protein is stimulated by growth factors and mitogen, thereby causing GEF aggregation in RAS protein, promoting RAS protein activation and activating downstream pathways. KRAS gene does matter considerably in regulating cell growth, proliferation, differentiation and apoptosis, the mutation of which inhibits the hydrolysis of RAS-GTP to RAS-GDP, and leads to the continuous activation of downstream pathways, thereby ultimately bringing about tumorigenesis 11.

Based on the degree of tissue structural disorder and nuclear abnormality, intraepithelial neoplasia of the pancreas (PanIN), a precancerous lesion of PDAC, is divided into three stages (I-III) from low to high 12. KRAS gene mutation is the initiating factor for the occurrence of PDAC, and over 90% of low-grade PanIN is coupled with KRAS gene mutation and shortened telomere corrosion 13, 14. In the PDAC gene engineered mouse model (GEMM), KRAS mutation and TP53 allele deletion jointly lead to the dedifferentiation and PanIN of pancreatic epithelial cells, while the loss of KRAS mutation results in the regression of lesions, indicating the decisive role of KRAS mutation in the development of PDAC 15. Besides, studies have also shown that a single KRAS gene mutation is not enough to maintain the concentration of activated KRAS protein that brings about PDAC. TP53 mutation can inhibit GAP activity and have more KRAS protein activated, thereby causing PDAC 16. At the same time, some studies have indicated that the larger the copy number of KRAS gene mutation is, the higher the malignant degree of the tumor becomes, which plays an important role in the substitution of low-dose KRAS mutated tumor MYC proto-oncogene (MYC), YAP and nuclear factor of kappa light polypeptide gene enhancer in B cells 2 (NFKB2), and promotes the invasion and metastasis of the tumor 17. To this end, the conclusion can be drawn that KRAS mutation is the initiating factor of PDAC, and that TP53, SMAD4 and other genes play an important synergistic role in the occurrence and development of PDAC.

## 2. Mutation Types of KRAS Gene and Their Relationship with PDAC Diagnosis and Prognosis

KRAS mutations in PDAC are mainly missense mutations, most observed in codon 12. According to the different bases of missense mutations, KRAS mutations can be divided into G12D, G12V, G12R, G12C, G12A and G12S, among which, G12D mutation is the most common one. G12D patients account for 51% of all mutations, while G12V, 32%; G12R, 12%, and all other phenotypes, less than 5% 13, 18.

Other mutations with a lower frequency may also occur on codons 11, 13, 61, and 146^19^. The prognosis of patients with KRAS gene mutation has been studied for a long time, and most study results show that compared with patients with KRAS wild-type, those with KRAS mutation are exposed to a worse prognosis 20. For patients with resectable or unresectable PDAC, the prognosis is different for those with different KRAS mutation subtypes, among which, the overall survival (OS) period of patients with G12D mutation is 6 months, significantly shorter than that of those with other KRAS mutation types (KRAS wild type 9 months, G12V 9 months, G12R 14 months, P = 0.003)^21^. For patients with unresectable PDAC, G12D mutation is also an independent risk factor for poor prognosis, while KRAS mutation Q61 subtype predicts a better survival 22, which may be mainly attributed to the subtle differences in downstream effector proteins and downstream pathways of KRAS mutations of different subtypes 23, 24.

Liquid biopsy technology is an emerging non-invasive pathological detection technology, and obtains tumor gene or protein expression in patients mainly by detecting the marker information released by tumor cells into body fluids. Circulating tumor DNA (ctDNA) is a portion of DNA released by tumor cells into the peripheral blood, with a short half-life, from 15 minutes to less than 2 hours, and is cleared quickly in the peripheral blood, making it a marker for monitoring tumor dynamics in real time. ctDNA is used to monitor efficacy, drug resistance and recurrence. Previous studies have shown that the ctDNA detection results for patients after PDAC are closely related to their prognosis, which can effectively predict tumor recurrence and metastasis, and stratify the risk of these patients. On the one hand, the specificity of ctDNA KRAS mutation is as high as 96% in the early diagnosis of PDAC, while the sensitivity remains only 30%. In combination with other protein biomarkers carbohydrate antigen 199 (CA199), carcinoembryonic antigen (CEA) and osteopontin can be increased to 64% 25. Therefore, ctDNA can assist the clinical more sensitive detection of abnormalities in the early stage of PDAC. On the other, KRAS mutations in ctDNA have also been used for predicting prognosis. Lee et al. 26 detected preoperative and postoperative ctDNA KRAS mutations in 38 patients with surgically resectable PDAC, and the results show that patients with KRAS positive ctDNA preoperatively experience a shorter median recurrence-free survival (RFS) (10.3 months, P=0.002) and a shorter median OS (13.6 months, P=0.015) compared with those without KRAS. Postoperative ctDNA-KRAS positive patients are exposed to a faster rate of recurrence, a shorter median RFS (5.4 months versus 17.1 months, P < 0.001), and a shorter median OS (10.6 months, P =0.003) than negative patients, indicating that positive ctDNA KRAS is closely related to poor prognosis. CtDNA variation can be detected by blood during the perioperative period, which thereby assists the judgment of clinical prognosis.

## 3. Advances in KRAS Targeting Drugs for Pancreatic Cancer

Currently, no KRAS inhibitor for PDAC has been approved, and KRAS was once considered drug-resistant. In turn, the KRAS gene is indirectly inhibited by inhibiting its upstream and downstream targets. With recent advances in research, direct inhibitors of KRAS gene in lung cancer have been approved for marketing. According to the location of inhibition targets, drugs acting on KRAS pathway can be divided into three categories, i.e., upstream KRAS molecular targeting drugs, KRAS targeted drugs and KRAS downstream molecular targeting drugs.

### 3.1 KRAS upstream molecular targeting agents

First-generation EGFR inhibitors, such as gefitinib and erlotinib, improve little in patients with PDAC either in combination with gemcitabine or with tigio, and the overall survival time (OS) is slightly lower in the gemcitabine plus erlotinib group than in the gemcitabine monotherapy group in Phase III clinical trial (LAP07) in advanced pancreatic cancer 27. A Phase II clinical study in Korea showed that the OS of the gemcitabine and tigio combined with erlotinib group was lower than that of the gemcitabine and tigio group 28, which is attributed to the compensatory expression of three other members of the human epidermal growth factor receptor family (HER), including four members, i.e., EGFR/HER1, ERBB-2 /HER-2, ERBB-3 /HER-3 and ERBB-4 /HER-4^29^. Inhibitors of both EGFR and ERBB, such as afatinib, have been frequently studied in lung cancer and been proven effective 30. A phase II clinical study of afatinib in patients with PDAC (NCT02451553) is currently underway.

Other upstream targets include son of sevenless 1 (SOS1), a GEF member that inhibits KRAS activation by preventing its binding to KRAS. Previous studies mainly focused on synthetic drugs directly binding SOS1 to prevent it from binding KRAS. Although these compounds can effectively bind SOS1, the blocking efficiency remains low 31, 32. New SOS1 inhibitors BAY293 33 and BI3406 34 have achieved a good inhibition effect on tumor cells, and further animal and human tests are needed for verification.

Protein tyrosine phosphatase (SHP2) is both a non-receptor protein tyrosinase and a scaffold adaptor binding to effector proteins^35^, which connects SOS1, growth factor receptor bound protein 2 (GRB2) and KRAS in the KRAS pathway, thereby mediating the activation of KRAS^36^. In 2016, the first selective oral SHP2 inhibitor SHP099 appeared 37. With the discovery of drug binding sites, an increasing number of SHP2 inhibitors, such as SHP389, BBP398, HBI2376, etc., have come out 38-40. No targeted inhibitors for SHP2 are currently approved for marketing, and most of the drugs are in Phase I and II clinical trials in patients with advanced solid tumors, including PDAC.

GRB2 is a 25kDa adaptor protein composed of one SH2 and two SH3 domains, which can bind to SOS1 for promoting the activation of KRAS, known as a potential therapeutic target for KRAS-related tumors 41. The high-affinity GRB2-binding peptides HAGBPs and PTD-GRB2-SH2 42 can block the connection between SH3 domain and SOS1, thus blocking the conduction of carcinogenic signal. Current research on GRB2 mainly focuses on hematologic tumor and breast cancer, and a Phase I clinical trial (NCT04196257) is underway for solid tumors such as PDAC.

### 3.2 KRAS molecular targeting agents

KRAS is the first discovered human tumor gene, and KRAS protein is equipped with a nearly spherical structure with no obvious binding site, which makes it difficult to synthesize a compound that can target binding and inhibit its activity. It was not until 2013 that Shokat and his team 43 discovered an allosteric binding pocket behind the KRAS-G12C protein switch-II. It was exactly the very discovery that led to the development of several small molecule covalent inhibitors targeting KRAS G12C mutations. Among them, Amgen's sotorasib(AMG510) was approved by the US Food and Drug Administration(FDA) in May 2021 for non-small cell lung cancer patients with KRAS G12C mutation, becoming the first KRAS-targeted drug hitting the world market. Sotorasib is a highly selective small-molecule inhibitor that targets KRAS-G12C and covalently binds to the switch-II allosteric binding pocket, which keeps KRAS-G12C inactive and inhibits the downward transmission of KRAS-oncogenic signals. It has shown promising results in Phase I and II trials in advanced solid tumors, with 8 of the 12 enrolled patients with advanced PDAC suffering from stable disease (SD) and 1 having a partial remission 44. Meanwhile, several KRAS G12C inhibitors are in Phase I/II clinical trials. Mirati Therapeutics' adagrasib is attracting extensive attention. MRTX1257, another KRAS G12C inhibitor of the company, is still in preclinical development. FDA has approved a clinical trial application developed by Wellspring Biosciences for ARS-3248, and Janssen Biotech will take over the Phase I clinical trial and subsequent development. Other KRAS-G12C targeted inhibitors for PDAC, such as GFH925 and JAB21822, are currently in Phase I or II clinical trials.

In addition to small molecule drugs directly targeting KRAS, other innovative therapeutic modalities for KRAS, such as RNA Interference (RNAi) therapies and KRAS-associated tumor vaccines, are also being extensively explored.

RNAi has been a hot research topic since it was first reported in 1998. In recent years, small interfering RNA (siRNA) has been frequently used for targeted therapy of PDAC patients, with an efficient delivery platform acting as the key to its clinical efficacy. Early delivery platforms were divided into viral vectors (adenoviruses and retroviruses) and non-viral vectors (cationic liposomes, etc.) 45. The extracellular matrix of pancreatic cancer is composed of dense connective tissue. Exosomes are extracellular vesicles containing mRNA and proteins involved in the transmission of information between cells 46. The CD47 transmembrane protein of exosomes helps to evade host immune clearance, making it an efficient delivery vector for siRNA 47. Exosome delivery siRNA targeting KRAS-G12D has presented superior efficacy in animal studies 48, and a Phase I clinical trial (NCT03608631) is currently underway.

A KRAS-associated cancer vaccine has emerged more than once. Currently, the most promising pancreatic cancer vaccine is GI4000, which expresses KRAS peptides of various mutant subtypes. In the Phase II clinical trial of KRAS-mutated lung cancer patients, 50% of them exhibited specific antigen-antibody reactivity 49. KRAS peptide vaccine is currently in Phase I/II clinical trials for patients with PDAC (NCT03329248), and Mdc3/8 dendritic cell vaccine targeting KRAS mutations is also currently undergoing Phase I clinical trials (NCT03592888) 50.

### 3.3 KRAS downstream molecular targeting agents

Downstream of KRAS involves multiple signal transduction pathways and more than 80 effector proteins, among which, the most studied and understood are RAF-MEK-ERK, MAPK cascade signaling, PI3K-AkT and Hipoo-YAP pathway. Specific inhibitors targeting these targets have been or are being tested in clinical trials.

RAF proteins, including ARAF, BRAF and CRAF, are key components of MAPK. RAF, the first kinase of this pathway, is considered an ideal target for the development of anticancer drugs 54. First-generation RAF inhibitors have been developed and used for BRAF (V600E) - carrying therapies, such as Vemurafenib 55, Dabrafenib 56, and Encorafenib 57. Good results have been obtained at the initial treatment stage, but tumor drug resistance remains the main problem existing at present. The main mechanisms are: (i) upregulation of KRAS activity, which brings about abnormal activation of ERK; and (ii) BRAF producing N-terminal truncated variants, and reducing drug affinity 58. The second generation of pan-RAF inhibitors, including LY3009120 59, TAK632 60, TAK580 61, CCT3833 62 and other drugs, can effectively avoid the above-mentioned problem. However, there are limited clinical data of both first-generation and second-generation RAF inhibitors in advanced solid tumors such as PDAC. A Phase II clinical trial (NCT04390243) is currently underway in PDAC patients in combination with the RAF inhibitor Encorafenib and the MEK inhibitor Binimetinib.

The most important members in the MEK family are MEK1 and MEK2, two key nodes in the MAPK level pathway. Currently, most of the inhibitors targeting MEK are non-ATP competitive inhibitors, which function by combining themselves with MEK hydrophobic region to inhibit phosphorylation 63. Currently, there are four MEK1/2 inhibitors on the market, i.e., Selumetinib 64, Binimetinib 65, Cobimetinib 66 and Trametinib 67. Other MEK inhibitors include Pimasertib. In the Phase I/II clinical trial (NCT01016483) of pimasertib combined with gemcitabine in patients with advanced PDAC, the OS and Progression Free Survival (PFS) of patients in the Pimasertib combined with gemcitabine group were found 0.33 and 0.2 months longer than those in the gemcitabine group alone, not statistically significant, which increased the incidence of adverse reactions 52. A similar Phase II trial of Trametinib in combination with gemcitabine (NCT01231581) also presented limited efficacy 53. The poor efficacy of MEK single drug also results from tumor drug resistance, and the current drug resistance mechanism includes reactivation of MAPK level pathway; up-regulation of other parallel pathways, such as PI3K, signal transducer and activator of transcription (STAT) and Hippo signaling pathway 68-70; and transformation of cell phenotypic 71. Attempts have been made on several combinations to address these resistance mechanisms, among which, MEK inhibitors combined with RAF inhibitors are approved by the FDA for the treatment of melanoma 72, 73. MEK inhibitors combined with PI3K inhibitors, AKI inhibitors, PI3K/mTOR dual inhibitors, YAP inhibitors and STAT3 inhibitors have all achieved good preclinical efficacy 69, 74-76. Although pancreatic cancer has not yet benefited from immunotherapy, MEK inhibitors have been proven to increase T cell infiltration in the tumor microenvironment and present significant synergies with immunotherapy such as programmed death-receptor 1 (PD-1) and programmed cell death-ligand 1 (PD-L1), compared with MEK inhibitors alone 77.

ERK is another target of MAPK-class pathway, with ERK1 and ERK2 included in the ERK family 78. Currently, there are no approved ERK1/2 inhibitors on the market, but some small molecule ERK inhibitors, such as GDC0944 79, Ulixertinib 80, CC90003 81 and other drugs, have already been under preclinical or clinical studies. ERK1/2 inhibitors can inhibit epithelial mesenchymal transition (EMT), up-regulate cellular markers of aging, and activate pancreatic stellate cell autophagy in pancreatic cancer.

Another important pathway for the occurrence and development of PDAC is the PI3K-Akt-mTOR pathway. PI3K/Akt/mTOR signal transduction pathway widely exists in tissue cells and is involved in the regulation of cell growth, proliferation, and differentiation. PI3K is the most important component of THE PI3K/AKt/mTOR signaling pathway. Activation can further phosphorylate phosphatidylinositol 4, 5-diphosphate (PIP2) into phosphatidylinositol 3,4, 5-triphosphate (PIP3). PIP3 acts as a second messenger to translocation AKt from the cell membrane to the cytoplasm, and activate configurational changes. Thus, downstream mTOR and other targets can be activated to regulate protein translation and cell growth. Abnormal activation of any of these targets is strongly associated with malignancy 82. Up to now, five PI3K inhibitors, including Idelalisib 83, Copanlisib 84, Duvelisib 85, Alpelisib 86 and Umbralisib 87, have been approved for marketing worldwide, which are mostly used for the treatment of breast cancer and hematological tumors, but have not been used for PDAC treatment. Preliminary studies have proved that PI3K inhibitors alone are not safe for PDAC patients, and their efficacy is limited. A Phase II/III trial of Rigosertib (a PI3K targeted inhibitor) in combination with gemcitabine in patients with advanced PDAC showed that Rigosertib failed to improve patient outcomes 88. Currently, no AKI targeted inhibitors have been approved for marketing, but inhibitors including AZD5363 89, GSK2141795 90 and other drugs have been or are undergoing clinical trials, with preliminary data showing limited efficacy of single agents. mTOR includes mTORC1 and mTORC2. The former regulates cell growth while the latter is related to cell survival and proliferation 91, 92. Already approved drugs for the treatment of renal cell carcinoma, lymphoma and other diseases include sirolimus, everolimus, tesirolimus, etc., and clinical trials are underway for patients with PDAC alone or in combination.

Hippo-YAP pathway is another important downstream effector pathway of KRAS, involved in cell proliferation, apoptosis, and control of organ size 93. The core of Hippo pathway is a kinase cascade reaction, in which MST1/2 kinase and SAV1 form a complex that phosphorylates and activates LATs1/2. LATs1/2 can further phosphorylate YAP/TAZ, and the phosphorylated YAP/TAZ cannot enter the nucleus and will be ubiquitinated and degraded in the cytoplasm, which cannot play its proliferation-promoting and anti-programmed cell death activities, either. If the MST1/2-YAP/TAZ pathway is blocked or inactivated, the unphosphorylated YAP/TAZ enters the nucleus and binds with downstream transcription factors such as transcriptional enhanced associate domain (TEAD) to promote the expression of target genes 94. Yiet al. 95 found that YAP plays a limited role in the first step of PDAC transformation, acino-ductal metaplasia (ADM), but a considerable role in the transformation of PanIN to PDAC. In addition, YAP can disrupt tumor-matrix interactions that promote PDAC invasion and metastasis 96. High expression of YAP is associated with poor prognosis of PDAC 97-99. At the same time, studies have shown that Hippo-YAP pathway is related to drug resistance of tumor cells to chemotherapy and targeted therapy 100, 101. Targeted blocking of the MAPK cascade signaling pathway will lead to the upregulation of Hippo pathway, thereby mediating the drug resistance of targeted drugs. Albumin-paclitaxel combined with gemcitabine is a first-line chemotherapy regimen widely applied to pancreatic cancer, but most patients rapidly develop drug resistance after several courses of treatment. Albumin paclitaxel is an anti-tubulin drug, and its action mechanism depends on the activation of cell cycle kinase CDK1 to induce apoptosis of cancer cells, while the mutation and upregulation of YAP targets can reduce the apoptosis inducing ability of anti-tubulin drugs 102, 103. The combination of PD-1 and PD-L1 can down-regulate the response of the immune system to human cells, regulate the immune system, and promote the tolerance by inhibiting the inflammatory activity of T cells. The expression of PD-1 and PD-L1 in tumor cells can lead to immune evasion, while the activation of YAP or TAZ can up-regulate the expression of PD-1 and PD-L1 in tumor cells, thereby mediating the immune escape of tumor cells 104-106.

The pathogenesis of the Hippo pathway mentioned above also provides a new treatment vision, i.e., YAP targeted inhibitors combined with chemotherapy, targeted and immunotherapy. Current YAP targeting inhibitors mainly include two types, i.e., inhibitors directly targeting YAP and those targeting YAP downstream transcription factors. Verteporfin is the only existing small molecule inhibitor that acts directly on YAP targets and can bind to YAP to block the interaction between YAP and TEAD 107. Nie et al. 108 have treated patients with KRAS mutation-positive pancreatic cancer using Verteporfin in combination with a pan-Raf targeted inhibitor (LY3009120), and the results show that the combination significantly enhances the anti-tumor efficacy of LY3009120; Roberge et al.^109^ found in animal studies that compared to gemcitabine alone, Verteporfin in combination with gemcitabine improves survival in mice, which, however, is photosensitive and cytotoxic and has been proven exposed to relatively large side effects^110^, ^111^. To this end, the downstream TEAD transcription factor targets such as Vinylsulfonamide derivatives have been studied instead 112.

### 3.4 KRAS gene mutation and autophagy

Cell autophagy is mediated by lysosomes in eukaryotes, the degradation of highly conservative in the energy shortage, reactive oxygen species under various stress conditions such as accumulation, cells that can pass autophagosome formation, the intracellular substances to the soluble enzyme degradation and recycling in the body, and thus obtains the material and energy needed for powering a cell survival 113. Compared with normal pancreatic duct cells, autophagy in PanIN and PDAC cells is up-regulated 114. However, the activation of the Raf-MEK-ERK pathway can inhibit autophagy 115, 116. The targeted inhibition of the Raf-MEK-ERK pathway can up-regulate autophagy and increase the PDAC cells' tolerance to autophagy. The combination of RAF-MEK-ERK pathway targeting inhibitors and autophagy inhibitors chloroquine or hydroxychloroquine can effectively inhibit the growth of PDAC cells 117-119. Several studies have initiated clinical trials of combined MAPK pathway targeting inhibitors and autophagy inhibitors, i.e., NCT04145297, NCT03825289, and NCT04132505. Not only can the inhibition of autophagy inhibit the growth of PDAC cells, but also excessive induction of autophagy can inhibit the proliferation of PDAC cells. MTOR regulates autophagy by sensing changes in nutrient levels inside and outside cells, and inhibits autophagy when activated 120. The mTOR targeting inhibitor Sirolimus can over-induce autophagy, thereby inhibiting the proliferation of PDAC cells 121.

### 3.5 KRAS gene mutation and pancreatic cancer microenvironment

The microenvironment of pancreatic cancer refers to the local environment of pancreatic cancer cells, involving cellular and non-cellular components. The former mainly include pancreatic stellate cells (PSC), cancer-associated fibroblasts (CAF), and immune cells, while the latter, also known as extracellular matrix (ECM), contain collagen, matrix proteins and a variety of soluble factors. Different from other solid tumors, pancreatic stroma accounts for more than 80% of the tumor volume and contains abundant extracellular matrix, pancreatic stellate cells and tumor-associated fibroblasts, known as one of the reasons for the poor response to chemoradiotherapy, targeted and immunotherapy^122^. Mutation of KRAS gene can alter the tumor microenvironment of pancreatic cancer by changing the composition of extracellular matrix after inducing the production of various chemokines and fibroblasts. It is widely acknowledged that Hedgehog signaling pathway matters considerably in embryonic development, stem cell regulation, etc., and is highly activated in PDAC. Besides, sonic hedgehog (SHH) is the ligand of hedgehog signaling pathway 123. Tape and colleagues found that KRAS-G12D can activate SHH, thereby promoting the secretion of multiple ECM 124. Targeted inhibition of SHH can promote the TME of pancreatic cancer, and thus improve the response of pancreatic cancer cells to chemotherapy. SHH inhibitors combined with chemotherapy have achieved good efficacy in animal experiments 125, 126. However, several clinical studies in patients with advanced PDAC have found that SHH-targeting inhibitor Vismodegib combined with gemcitabine or paclitaxel fails to improve the OS and PFS in patients with PDAC 127, 128. The combination of saridegib, another SHH inhibitor, with gemcitabine, results in a higher incidence of PDAC progression than placebo and gemcitabine monotherapy 129.

To sum up the above progress of cancer-pathway targeting drugs in upstream and downstream KRAS, it is found that whether for longitudinal administration of AKT inhibitor in combination with PI3K inhibition, or for horizontal administration of BRAF inhibitor in combination with mTOR inhibitor 130, or vertical administration of downstream targeted in combination with upstream and middle targets, the clinical efficacy is superior to that of single administration and can achieve more lasting antitumor effect. Inhibitors simultaneously inhibiting multiple target sites, such as PI103 131, PKI587 132 and PKI179 133, known as dual targeting inhibitors of mTOR and PI3K, are also being developed. Studies have also shown that targeted inhibition of the PI3K-AKT-mTOR pathway can prevent cell repair of DNA damage, thereby making patients more sensitive to radiotherapy and chemotherapy 134. This finding has been used to treat head and neck malignancies 135, 136. Besides, inhibition of PI3K-AKT-mTOR pathway in pancreatic cancer cells can inhibit cancer-associated fibroblasts (CAF), reduce interstitial fibrosis, and alter pancreatic cancer tumor microenvironment (TME), which will thereby increase the lethality of chemotherapy drugs 137. Therefore, combined radiotherapy and immunotherapy, not only the combination of targeted drugs with different targets, but also that of targeted drugs with chemotherapy, are both the direction for future research 138. However, the underlying mechanisms for the combination therapy remain unclear, and avoiding the accumulation of toxic effects of drug combination on normal cells is still a tough challenge in the future 139.

## 4. Summary and Outlook

In summary, the clinical value of KRAS gene in the treatment of PDAC is beginning to dawn. On the one hand, the role of KRAS mutations in the occurrence and development of PDAC has been basically clear, while on the other, the medicinal properties of KRAS gene have achieved a breakthrough in solid tumors such as lung cancer and colorectal cancer. Although the mutation rate of KRAS G12C is less than 5% in Chinese lung cancer population while that in PDAC is less than 1% 140, it still brings promising treatment efficacy to the patients, which seems to open a new pattern of clinical research on KRAS mutated tumors. In addition to targeted drugs, Sotorasib is found to alter TME in mice, promote T cell infiltration, and produce pro-inflammatory TME, thereby producing long-lasting anti-tumor effects alone or in combination with immune checkpoint inhibitors. Besides, it is widely acknowledged that the immunotherapy of PDAC progresses slowly. Studies have found that PDAC lacks CD8+T cell infiltration, and the high infiltration of myeloid suppressor cells and tumor-associated macrophages results in PDAC alone not being sensitive to anti-PD-L1 therapy. Most clinical trials have presented poor immunotherapy effect, and no obvious efficacy has been observed. Therefore, PDAC is recognized as a "cold tumor". In this case, whether KRAS-targeted drugs can also bring a breakthrough in immunotherapy for PDAC remains to be seen. Finally, it is also noteworthy that the drug resistance of targeted drugs in tumor therapy should not be ignored. In June 2021, the drug resistance mechanism of Adagrasib was published on the *New England Journal*, which involves secondary mutation or amplification of KRAS, bypass activation, other driver mutations, and histopathological transformation, etc. 141. Studies have been conducted to explore these mechanisms and solutions concerning the acquired resistance to KRAS inhibitors 142.

It is still expected that with the technological progress and the continuous accumulation of clinical practice, an increasing number of biomarkers, signaling pathways and targets can be found for the individualized diagnosis and treatment of PDAC, and ultimately benefit the related patients.

## Figures and Tables

**Figure 1:**
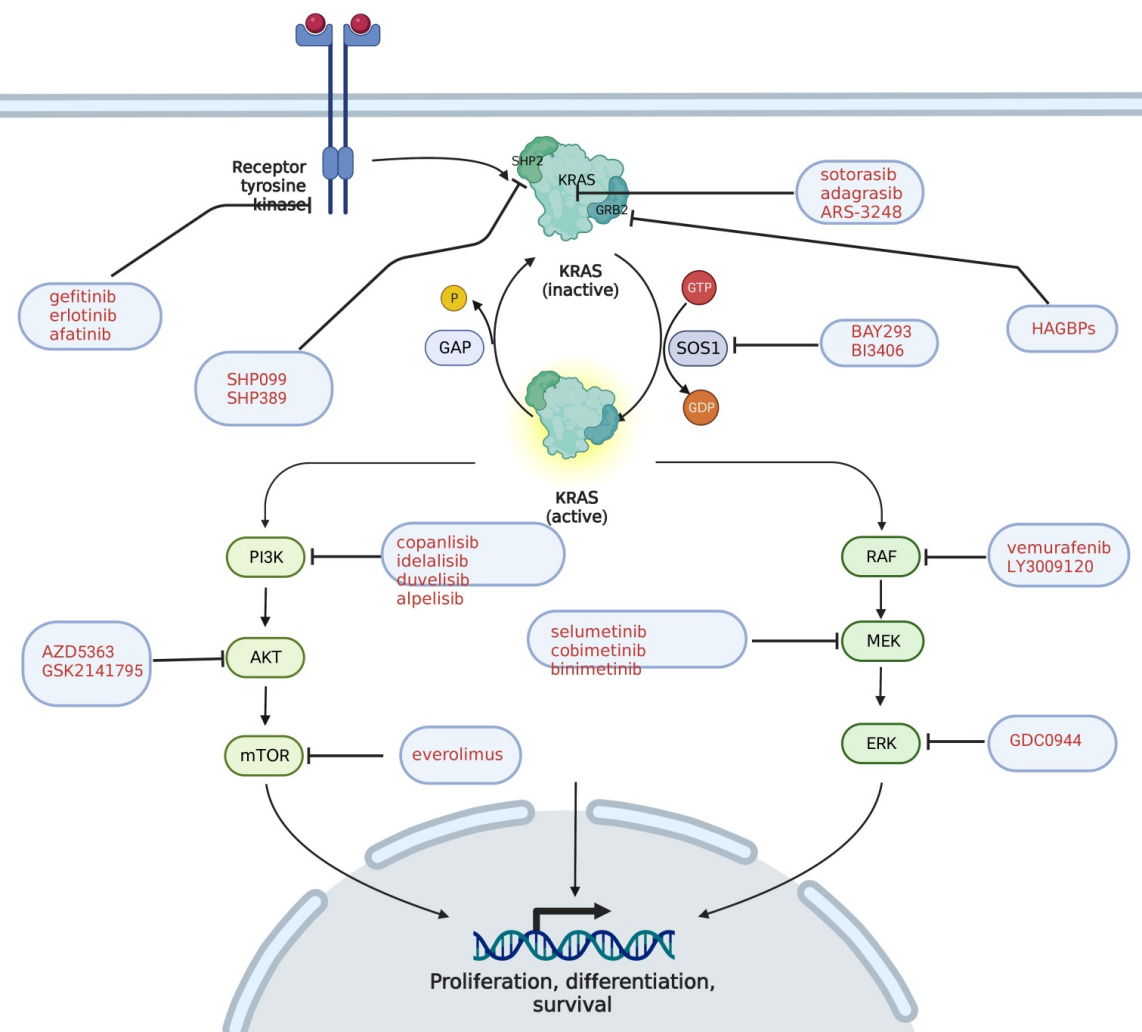
** KRAS pathway and its targeted inhibitors.** EGFR transmits signals to KRAS and activates KRAS; GRB2 promotes the activation of KRAS; and SHP2 is a scaffold protein that connects KRAS, GRB2 and SOS1. The red text represents the targeted inhibitor for this target.

**Figure 2:**
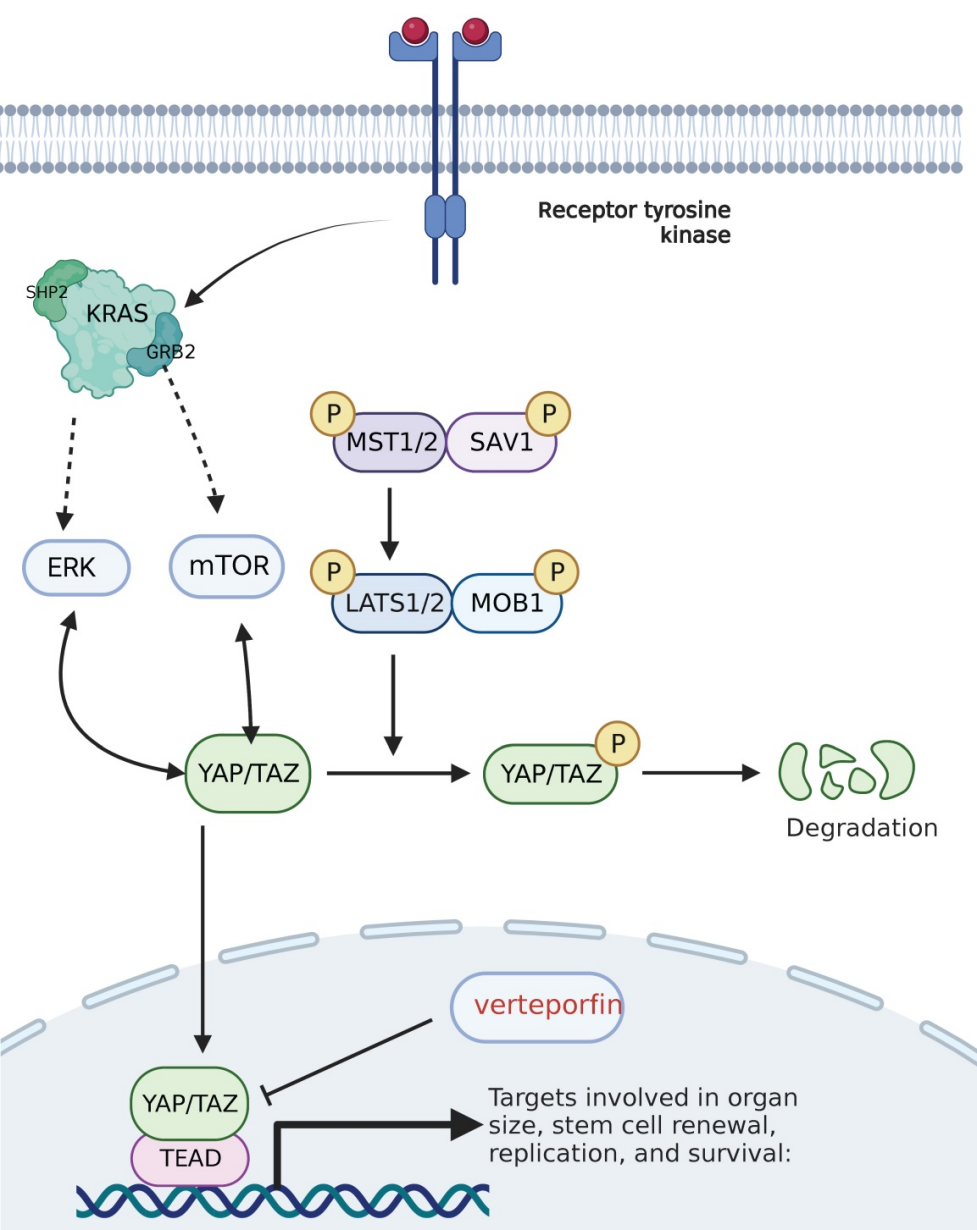
** Hippo pathway and its targeted inhibitors.** Mst1/2 kinase and SAV1 form a complex that phosphorylates and activates LATs1/2; Lats1/2 kinase in turn phosphorylates and inhibits the transcription coactivators YAP and TAZ; and after dephosphorylation, YAP/TAZ is transferred to the cell nucleus and activates transcription factors such as TEAD1-4, thereby inducing and promoting cell proliferation, and inhibiting apoptosis. The red text represents the targeted inhibitor for this target.

**Table 1:** Single-target clinical trials

Target	Disease	Agent	Phase	Clinical Trial Number	Status/Results
EGFR	PDAC	Erlotinib	II	NCT01608841	51
EGFR	PDAC	Afatnib	II	NCT02451553	Active
GRB2	Solid tumor	BP1001	I	NCT04196257	Recruiting
SHP2	Solid tumor	HBI2376	I	NCT05163028	Recruiting
KRAS-G12C	Solid tumor	sotorasib	III	NCT03600883	44
KRAS	PDAC	Rat sarcoma KRAS vaccine	I	NCT05013216	Active
KRAS	PDAC	mDC318 KRAS vaccine	I	NCT03608631	Recruiting
KRAS-G12D	PDAC	exosome-KRAS(G12D)-siRNA	I	NCT03608631	Recruiting
KRAS	Solid tumor	MRTX849	I、II	NCT03785249	Recruiting
BRAF	PDAC	Vemurafenib	II	NCT05068757	Recruiting
MEK	Solid tumor	Pimasertib	I、II	NCT01016483	52
MEK	Solid tumor	GSK1120212	II	NCT01231581	53
ERK	Solid tumor	Ulixertinib	I	NCT04566393	Active
mTOR	PDAC	Sirolimus	I、II	NCT03662412	Recruiting

**Table 2:** Clinical trials of drug combinations

Target	Disease	Agent	Phase	Clinical Trial Number	Status/ Results
KRAS-G12C+SOS1	PDAC	MRTX849+BI1701963	I	NCT049752356	Recruiting
ERK+SHP2	PDAC	LY3214996+RMC4630	I	NCT04916236	Active
ERK+autophagy	Solid tumor	Binimetinib+ hydroxychloroquine	I	NCT04132505	Recruiting
KRAS-G12C+SHP2	PDAC	MRTX849+TN0155	I、II	NCT04330664	Active
ERK+BRAF	PDAC	Binimetinib+Encorafenib	II	NCT04390243	Recruiting
MEK+autophagy	PADC	Trametinib+ hydroxychloroquine	I	NCT03825289	Recruiting
CDK4/6+PI3K	PDAC	Palbociclib+Gedatolisib	I	NCT03065062	Recruiting
PI3K+mTOR	Solid tumor	Alpelisib+Everolimus	I	NCT02677933	Recruiting
